# Pseudomonas aeruginosa* gshA* Mutant Is Defective in Biofilm Formation, Swarming, and Pyocyanin Production

**DOI:** 10.1128/mSphere.00155-18

**Published:** 2018-04-18

**Authors:** Tricia A. Van Laar, Saika Esani, Tyler J. Birges, Bethany Hazen, Jason M. Thomas, Mamta Rawat

**Affiliations:** a Department of Biology, California State University—Fresno, Fresno, California, USA; University of Kentucky

**Keywords:** *Pseudomonas aeruginosa*, biofilms, glutathione, pyocyanin, thiols, virulence

## Abstract

Pseudomonas aeruginosa is a ubiquitous Gram-negative bacterium that can cause severe opportunistic infections. The principal redox buffer employed by this organism is glutathione (GSH). To assess the role of GSH in the virulence of P. aeruginosa, a number of analyses were performed using a mutant strain deficient in *gshA*, which does not produce GSH. The mutant strain exhibited a growth delay in minimal medium compared to the wild-type strain. Furthermore, the *gshA* mutant was defective in biofilm and persister cell formation and in swimming and swarming motility and produced reduced levels of pyocyanin, a key virulence factor. Finally, the *gshA* mutant strain demonstrated increased sensitivity to methyl viologen (a redox cycling agent) as well as the thiol-reactive antibiotics fosfomycin and rifampin. Taken together, these data suggest a key role for GSH in the virulence of P. aeruginosa.

**IMPORTANCE** Pseudomonas aeruginosa is a ubiquitous bacterium that can cause severe opportunistic infections, including many hospital-acquired infections. It is also a major cause of infections in patients with cystic fibrosis. P. aeruginosa is intrinsically resistant to a number of drugs and is capable of forming biofilms that are difficult to eradicate with antibiotics. The number of drug-resistant strains is also increasing, making treatment of P. aeruginosa infections very difficult. Thus, there is an urgent need to understand how P. aeruginosa causes disease in order to find novel ways to treat infections. We show that the principal redox buffer, glutathione (GSH), is involved in intrinsic resistance to the fosfomycin and rifampin antibiotics. We further demonstrate that GSH plays a role in P. aeruginosa disease and infection, since a mutant lacking GSH has less biofilm formation, is less able to swarm, and produces less pyocyanin, a pigment associated with infection.

## Abstract (precis)

Pseudomonas aeruginosa is a ubiquitous bacterium that can cause severe opportunistic infections, including many hospital-acquired infections. It is also a major cause of infections in patients with cystic fibrosis. P. aeruginosa is intrinsically resistant to a number of drugs and is capable of forming biofilms that are difficult to eradicate with antibiotics. The number of drug-resistant strains is also increasing, making treatment of P. aeruginosa infections very difficult. Thus, there is an urgent need to understand how P. aeruginosa causes disease in order to find novel ways to treat infections. We show that the principal redox buffer, glutathione (GSH), is involved in intrinsic resistance to the fosfomycin and rifampin antibiotics. We further demonstrate that GSH plays a role in P. aeruginosa disease and infection, since a mutant lacking GSH has less biofilm formation, is less able to swarm, and produces less pyocyanin, a pigment associated with infection.

## INTRODUCTION

Pseudomonas aeruginosa, a Gram-negative bacterium, is responsible for opportunistic infections and is capable of causing both acute and chronic infections. P. aeruginosa is capable of surviving in human-associated environments with minimal nutritional availability (1). Importantly, over the last few decades, there has been a steady increase in the number of drug-resistant P. aeruginosa strains (2, 3). In the hospital setting, patients with surgical wounds or burns, patients fitted with catheters, or those assisted by mechanical ventilation are potentially at risk for life-threatening untreatable infections. Infections with P. aeruginosa are particularly problematic in patients with cystic fibrosis (4). The persistence of chronic P. aeruginosa lung infections in cystic fibrosis patients is attributed to biofilm formation, which enhances its adhesion to cell walls and enables it to evade host immune functions (5). Biofilm formation also facilitates antibiotic tolerance relative to free-living planktonic cells and accordingly limits eradication. Motility is also strongly associated with P. aeruginosa pathogenesis, as it enables colonization of different environments, attachment to surfaces, and formation of biofilms. P. aeruginosa bacteria are capable of twitching, swimming, swarming, and sliding motility. Swarming motility on semisolid surfaces (0.3 to 0.5%), in particular, represents a complex adaptation to stress conditions, such as nitrogen limitation (6, 7). Another set of P. aeruginosa virulence genes are involved in biosynthesis of phenazines, redox active metabolites that can act as reductants for molecular oxygen and ferric ions, participate in primary energy metabolism, are involved in cell-to-cell communication, and serve as antimicrobial agents (8). The blue-green phenazine, pyocyanin, is a key virulence factor that can kill competing organisms as well as host cells and can inactivate catalases to protect against reactive oxygen species (ROS) generated by host tissues (8).

In P. aeruginosa, virulence and biofilm formation are controlled by a hierarchical quorum-sensing (QS) system mediated by the two chemically distinct classes of signal molecules, the *N*-acylhomoserine lactones (AHLs) and the 4-quinolones. The *N*-(3-oxododecanoyl) homoserine lactone, a product of LasI, binds to LasR, activating transcription of many genes, including *rhlR*, which encodes a second quorum-sensing receptor. Since LasR activates expression of *rhlR*, disruption of *lasR* reduces expression of both LasR- and RhlR-regulated target genes. The 4-quinolones include the signal molecule 2-heptyl-3-hydroxy-4-quinolone, also known as pseudomonas quinolone signal (PQS). PQS controls its own production and packaging into membrane vesicles that deliver antimicrobial agents and toxins, and PQS has been implicated in iron acquisition (9).

Glutathione (GSH) is a low-molecular-weight tripeptide thiol present in eukaryotes and many bacteria, including P. aeruginosa (10). In most GSH-containing bacteria, the *gshA*-encoded γ-glutamylcysteine synthetase ligates the amino group of cysteine to the γ-carboxyl group of glutamate. In turn, the *gshB*-encoded GSH synthetase condenses the resulting γ-glutamylcysteine with glycine to generate GSH (11). In other GSH-containing organisms, a single enzyme, GshF, is able to catalyze both reactions.

A role for GSH in pathogenesis has been demonstrated in *Salmonella* lacking *gshA*, where the mutant disrupted in this gene was attenuated in the acute model of salmonellosis (12). Furthermore, Listeria monocytogenes mutants with defective GSH synthesis exhibited 150-fold attenuated infectivity in mice (13). These virulence features of GSH in L. monocytogenes may relate to its allosteric binding under infection conditions to PrfA, a master transcriptional regulator shown to induce expression of numerous virulence factors (14). Whether GSH is involved in virulence in P. aeruginosa is not clear. In a screen for P. aeruginosa PA14 genes involved in virulence in Caenorhabditis elegans, both *gshA* and *gshB* transposon mutants demonstrated attenuated infectivity (15). Murine models provided some conflicting results: Skurnik et al. found that a *gshA* transposon mutant was capable of dissemination in a neutropenic mouse model but had no difference in the ability to colonize the gastrointestinal (GI) tract (16), while Turner et al. found an increased abundance of a *gshA* transposon mutant in acute and chronic wounds (17).

In this study, we report on a *gshA* transposon mutant of P. aeruginosa lacking GSH and we demonstrate that this mutant is sensitive to oxidative stress, thiol-reactive antibiotics, impaired in biofilm formation and swarming, and has reduced levels of pyocyanin—features that collectively suggest a virulence role for GSH in P. aeruginosa.

## RESULTS

### The *gshA* transposon mutant has no detected glutathione production.

**FIG 1:**
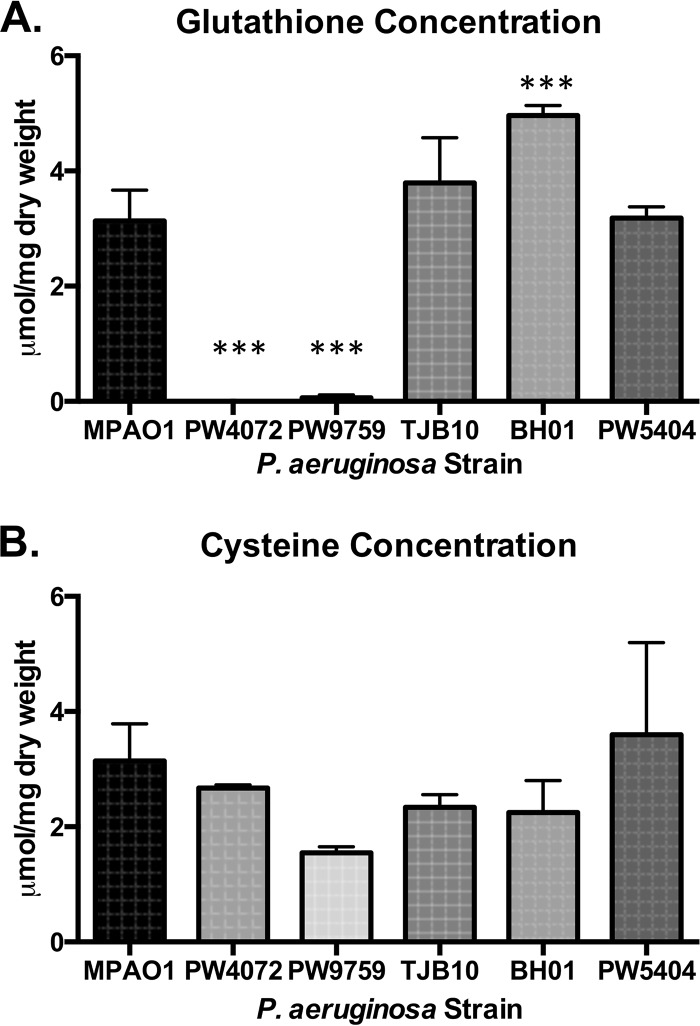
The *gshA* transposon mutants, strains PW4072 and PW9759, produce little to no detected glutathione. Glutathione (A) and cysteine (B) levels were quantified by HPLC. (A) The levels of glutathione were significantly decreased in the two mutant strains compared to the level in the wild-type strain (MPAO1). Additionally, there was a significant increase in glutathione production in the *cis-*complemented strain, BH01. (B) There was no significant difference in cysteine production among the strains tested. Values are means plus standard deviations (error bars) from four independent experiments. Values that are significantly different (*P* < 0.001) are indicated by three asterisks.

We obtained two *gshA* transposon mutants. In the first (PW4072), the transposon is inserted 843 bp from the 5′ end in open reading frame (ORF) PA5203, which is a single gene transcription unit. PW4072 was used to generate the *gshA* complemented strains TJB10 and BH01. In the second (PW9759), the transposon is inserted 1,147 bp from the 5′ end of PA5203. For an isotype control, we used strain PW5404 with a transposon inserted in PA2634, an isocitrate lyase. Thiol analysis of all strains was performed by high-performance liquid chromatography (HPLC). The wild-type, complemented, and isotype control strains all produced similar levels of GSH (3.13 ± 0.53, 3.79 ± 0.79, and 3.18 ± 0.20 μmol/mg, respectively) with the exception of BH01, the overexpression strain, which produced significantly more GSH than all strains (4.96 ± 0.18 μmol/mg), whereas the two transposon mutants, PA4072 and PW9759, produced little to no detected GSH (Fig. 1A). These results indicate that *gshA* is necessary for GSH production in P. aeruginosa and that restoration of *gshA* is sufficient for reestablishing GSH to normal levels. The levels of cysteine were not statistically different between the mutants and other strains (Fig. 1B). The levels of γ-glutamylcysteine, the precursor to GSH, were 0.38 ± 0.80 µmol/mg in the wild-type strain, 0.36 ± 0.07 µmol/mg in the complemented strain (TJB10), and was not detected in either *gshA* mutant.

### A lack of GSH leads to a decreased growth rate.

**FIG 2:**
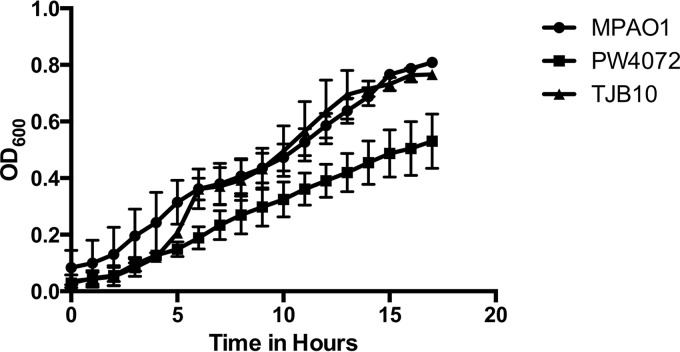
The *gshA* transposon mutant (PW4072) has a decreased growth rate compared to the wild-type (MPAO1) and complemented (TJB10) strains. Overnight cultures of each strain were grown in tryptic soy broth (TSB) supplemented with the appropriate antibiotics (tetracycline [60 µg/ml] and chloramphenicol [10 µg/ml] for strain PW4072; spectinomycin [200 µg/ml] for strain TJB10). A 250-µl portion of the overnight culture was used to inoculate 5 ml of fresh M9 minimal medium. Once the culture was grown to an OD_600_ of 0.5, each culture was diluted to an OD_600_ of 0.05 and inoculated into the wells of a 96-well plate. The cultures were grown with shaking at 37°C, and the OD_600_ was measured every hour for 18 h. Each strain was tested in triplicate.

When the strains were grown in minimal medium (M9), the *gshA* transposon mutant (PW4072) was defective for growth during exponential phase compared to strains MPAO1 and TJB10, exhibiting a roughly 1.6-fold reduction in cell number after 17 h of growth (Fig. 2). This growth impairment for strain PW4072 was not observed when the strains were grown in more complex media such as Luria-Bertani broth (LB) or tryptic soy broth (TSB) (data not shown), supporting the conclusion that when the P. aeruginosa cells are stressed by nutritional restriction, GSH is critical for maintaining cellular functionality.

### Disruption of *gshA* leads to decreased biofilm formation.

**FIG 3:**
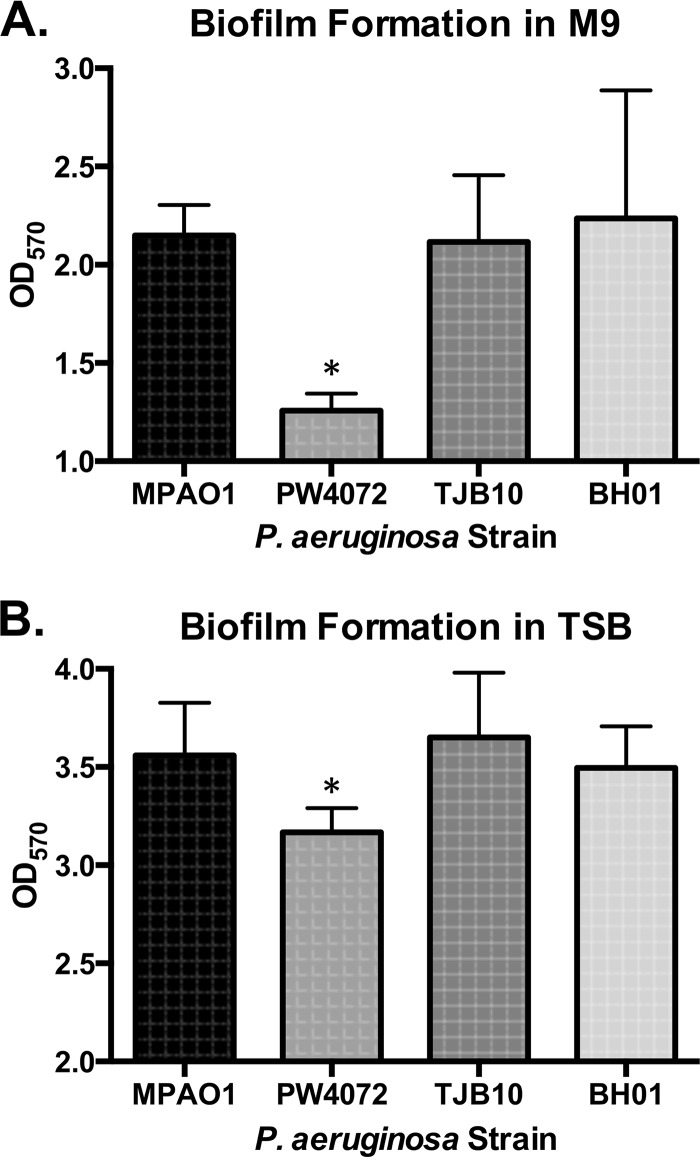
The *gshA* transposon mutant is defective for biofilm formation compared to the wild-type and complemented strains. Biofilms were grown in 96-well plates for 24 h in M9 medium (A) and TSB (B). Biofilms were rinsed three times with PBS, treated with 0.4% crystal violet for 15 min, rinsed again three times with PBS, and resuspended in 33% acetic acid. Biomass was read using a spectrophotometer at 550 nm. Values are means plus standard deviations (error bars) from four independent experiments. Values that are significantly different (*P* < 0.05) are indicated by an asterisk.

As GSH has been shown to be important for potassium transport in biofilms (18) and GSH biosynthesis genes are upregulated in biofilms of Candida albicans (19), we assessed whether GSH deficiency could alter biofilm formation in P. aeruginosa. Strains were grown for 24 h in minimal media in a 96-well plate after which biofilm biomass was assayed using crystal violet. As shown in Fig. 3A, strain PW4072 produced significantly less biomass (optical density at 570 nm [OD_570_] of 1.26) than strains MPAO1 (OD_570 _of 2.15; *P* = 0.042), TJB10 (OD_570 _of 2.49; *P* = 0.037), and BH01 (OD_550 _of 2.24; *P* = 0.027) did. Since we observed a growth defect in strain PW4072 when grown in M9 medium planktonically (Fig. 2), we also tested biofilm formation of all four strains in TSB, a medium in which PW4072 was able to grow at a normal rate. As shown in Fig. 3B, there was a decrease in biofilm formation in strain PW4072 compared to the wild-type strain (MPAO1) on TSB medium but not as much as that observed in minimal medium. This decrease was significant compared to the wild-type MPAO1 strain (*P* = 0.02) and complemented strains TJB10 (*P* = 0.002) and BH01 (*P* = 0.032), indicating that the lack of GSH contributes to decreased biomass production in the *gshA* transposon mutant. Restoration of GSH production resulted in restoration of biofilm formation.

### Glutathione contributes to swimming and swarming, but not twitching, motility in P. aeruginosa.

**FIG 4:**
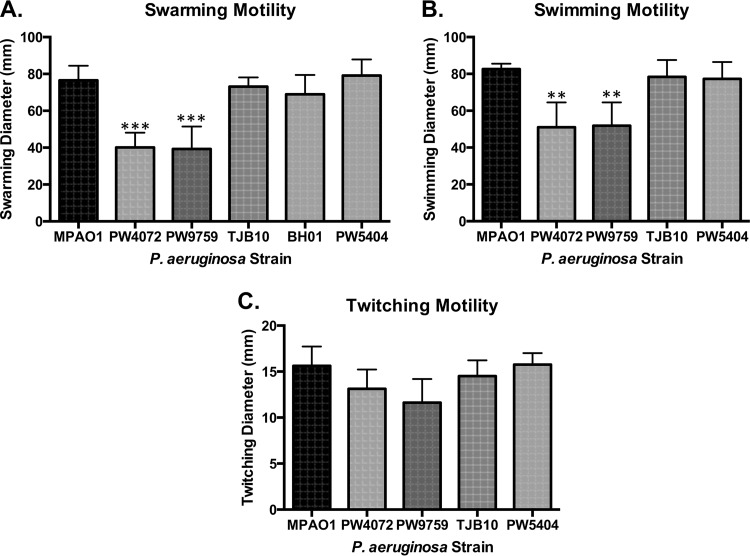
A lack of glutathione contributes to decreased swarming and swimming motility but has no effect on twitching motility in *gshA* mutant strains (PW4072 and PW9759). (A and B) Swarming and swimming motility was assessed for each strain by inoculating 3 µl of a 16-h overnight culture onto appropriate plates and incubating for 24 to 48 h at 30°C. (C) Twitching motility was assessed by stabbing an overnight culture through twitching agar and incubating for 24 to 48 h at 30°C. Data show decreased swarming and swimming motility of the mutant strains based on measured diameter. Values are means plus standard deviations (error bars) from four independent experiments. Values that are significantly different from the value for the wild-type MPAO1 strain are indicated by asterisks as follows: **, *P* < 0.01; ***, *P* < 0.001.

As motility is an important virulence factor for P. aeruginosa (20), we characterized how a disruption in the redox status would affect its motility. We tested three types of motility: swarming, swimming, and twitching. The mutant strains, PW4072 and PW9759, were noticeably defective for swarming motility compared to the wild-type strain (*P* = 0.0003 and *P* = 0.0002, respectively), with recovery of swarming in strains TJB10 and BH01 (Fig. 4A). There was no significant difference in swarming between the wild-type, complemented, and control strains. Although swarming motility was not reduced as significantly as is seen with defects in P. aeruginosa autoinducers (20) or flagellar motors (21), swarming motility was negatively impacted by the lack of GSH in the mutant strain. A similar trend was seen with swimming motility, where the PW4072 and PW9759 strains showed significant decreases in the ability to swim compared to the wild type (*P* = 0.004 and *P* = 0.005, respectively) (Fig. 4B), although the impairment was not as striking as observed with swarming motility. The wild-type, complemented, and control strains demonstrated similar swimming motility. We saw no significant differences in twitching motility among all the strains tested (Fig. 4C).

### Pyocyanin production is reduced when glutathione is lacking.

**FIG 5:**
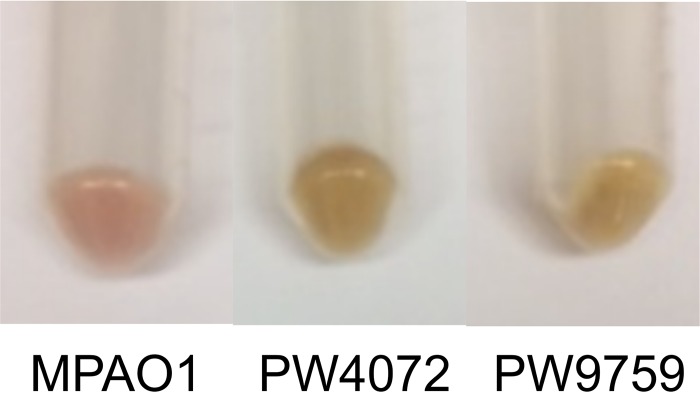
The *gshA* transposon mutants (PW4072 and PW9759) have different pigments than the wild-type strain (MPAO1).

**FIG 6:**
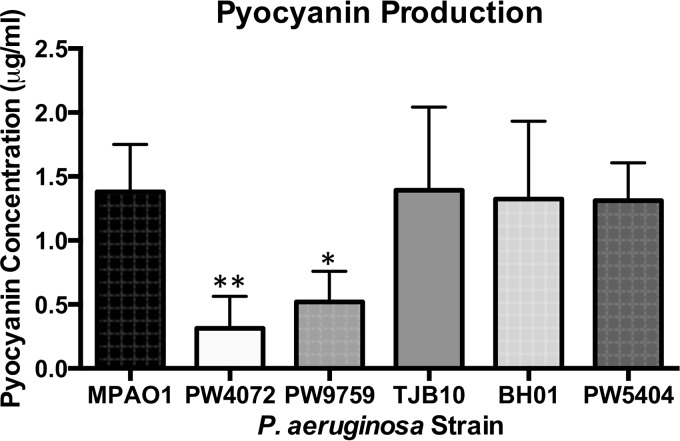
The *gshA* transposon mutants (PW4072 and PW9759) are deficient in pyocyanin production. Spent medium supernatants of cultures of each strain grown overnight in TSB were extracted with chloroform and HCl, and absorbance readings were taken at 520 nm for pyocyanin quantification. There was significantly less pyocyanin production in the *gshA* transposon mutant strain compared to the wild-type (MPAO1), complemented (TJB10 and BH01), and control (PW5404) strains. Values are means plus standard deviations from five independent experiments. Values that are significantly different from the value for the wild-type MPAO1 strain are indicated by asterisks as follows: *, *P* < 0.05; **, *P* < 0.01.

When *gshA* transposon mutants were grown in TSB medium, the cultures looked very different in color, with the *gshA* mutants, strains PW4072 and PW9759, being distinctly more yellowish and less green in color than strain MPAO1. The complemented strain was intermediate in color. Upon centrifugation, the pellets of the mutants were beige in color, while the color of the wild-type pellet was more pinkish (Fig. 5). Since pyocyanin is an important virulence factor produced by P. aeruginosa that imparts a distinct bluish-green color (22), we determined the pyocyanin levels in the different strains. The amount of pyocyanin produced by strains PW4072 (0.31 µg/ml) and PW9759 (0.52 µg/ml) was significantly lower than that produced by MPAO1 (1.24 µg/ml) (*P* = 0.008 and *P* = 0.046, respectively). Strains TJB10 (1.39 µg/ml) and BH01 (1.32 µg/ml) demonstrated recovery of pyocyanin production (Fig. 6). There was no significant difference in pyocyanin production between the wild-type and complemented strain. As with biofilm formation and swarming motility, pyocyanin production was not completely abolished with the disruption of GSH biosynthesis, although the levels were significantly decreased.

### Glutathione disruption does not inhibit quorum sensing mediated by acyl homoserine lactones.

**FIG 7:**
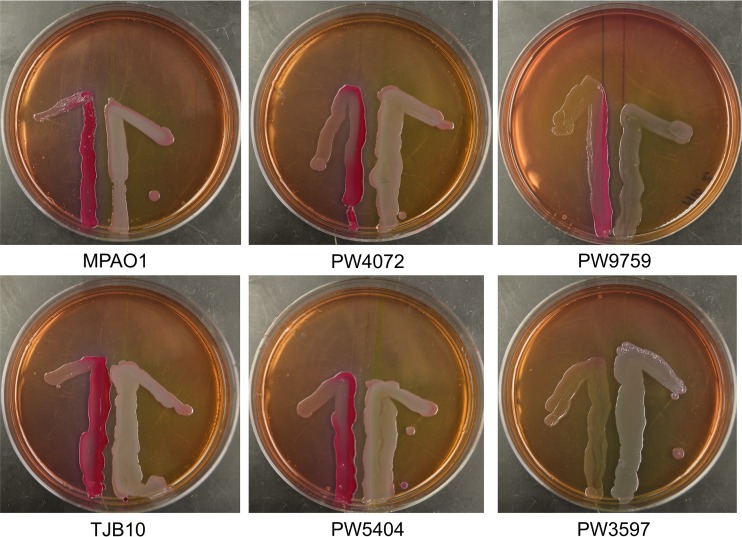
Glutathione disruption does not inhibit quorum sensing (QS) mediated by acyl homoserine lactones (AHLs). QS mediated by AHL was assayed using a cross-feeding assay. The P. aeruginosa strain of interest was streaked 0.75 cm from an E. coli reporter strain on MacConkey agar. The plates were incubated at 37°C for 24 h. In each image, the E. coli strain is on the left, while the P. aeruginosa strain is on the right. Strain PW3597 is defective for QS and serves as a negative control. All strains except PW3597 were positive for AHL-mediated QS as indicated by the pinkish color of the E. coli after 24 h of incubation.

QS is an important phenomenon known to be responsible for many aspects of P. aeruginosa virulence, including phenazine production, biofilm formation, and swarming motility. As we observed defects in these virulence factors in strains without GSH, we examined whether AHL-dependent QS was defective in the *gshA* transposon mutant strains. We used Escherichia coli strain MG4 containing plasmid pKDT17 as our reporter strain. pKDT17 contains a *lasB-lacZ* fusion coupled to *lasR*. In the presence of AHLs, *lasR* will initiate the transcription of LasB-LacZ fusion protein in strain MG4 resulting in β-galactosidase, whose activity would lead to purple/pink colonies on MacConkey agar. *E*. *coli* MG4 was streaked next to the P. aeruginosa strains of interest and observed after 24 h of incubation. A color change was seen when *E*. *coli* MG4 was placed next to all P. aeruginosa strains (Fig. 7), with the exception of the *lasR* transposon mutant (PW3597), which does not produce AHLs and served as a negative control. These results indicate that the *gshA* mutant strains are not defective for AHL and that AHL-dependent QS does not account for the impairment in biofilm formation, motility, or pyocyanin production observed.

### The *gshA* mutant is defective for persister cell formation.

**FIG 8:**
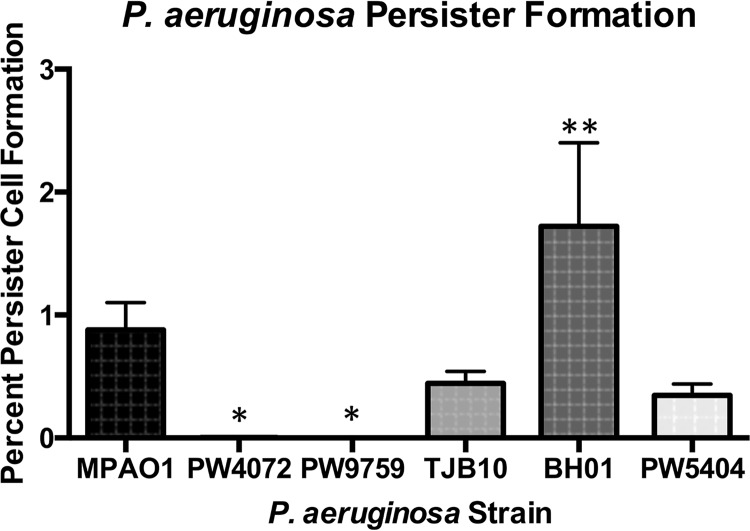
Persister cell formation is defective in *gshA* transposon mutants (PW4072 and PW9759). Persister cells were formed by exposing one stationary-phase culture to 50 µg/ml ofloxacin for 3.5 h. The number of CFU of untreated cultures was compared to that of ofloxacin-treated cultures to calculate the percentage of persister cells formed. The two mutant strains (PW4072 and PW9759) produced few to no persister cells, while the *cis-*complemented strain (BH01) produced significantly more persister cells than the wild-type strain (MPAO1). Values are means plus standard deviations from four independent experiments. Values that are significantly different from the value for the wild-type MPAO1 strain are indicated by asterisks as follows: *, *P* < 0.05; **, *P* < 0.01.

P. aeruginosa forms persister cells in response to various stressors, including antibiotic therapy, starvation, and reactive oxygen species (ROS) (23). Persister cells are a subpopulation of the overall population and are associated with chronic infections that fail to respond to antibiotic therapy (24). As production of ROS can be induced by various antibiotics (25), we wanted to determine how the lack of GSH would affect persister cell formation in P. aeruginosa. We induced persister cell formation in stationary-phase cultures of P. aeruginosa using the fluoroquinolone antibiotic ofloxacin and determined the percentage of persisters compared to an untreated population. As shown in Fig. 8, strains PW4072 and PW9759 produced significantly fewer persister cells (0.0025% [*P* = 0.02] and 0% [*P* = 0.02], respectively) than strain MPAO1 (0.65%) did. Strain TJB10 (0.43%) was not significantly different from MPAO1; however, strain BH01 (1.72%) produced significantly more persister cells than MPAO1 did (*P* = 0.0004).

### The *gshA* mutant displays increased sensitivity to methyl viologen.

**FIG 9:**
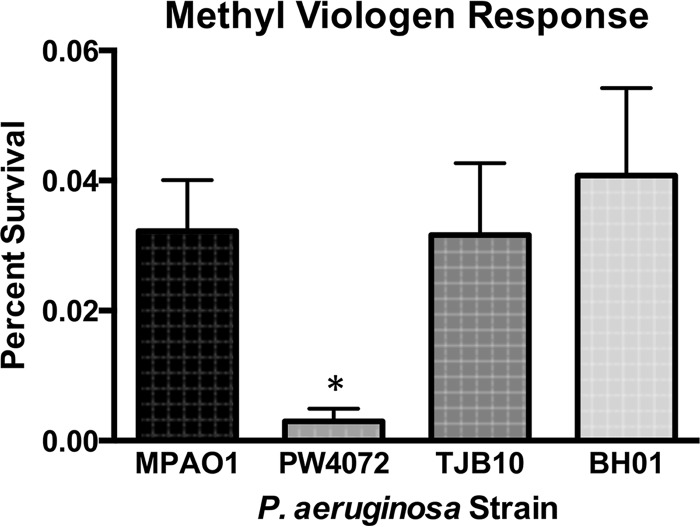
The *gshA* transposon mutant (PW4072) has increased sensitivity to methyl viologen. Strains were grown to exponential phase and plated on tryptic soy agar (TSA) alone or supplemented with 1.0 mM methyl viologen. The surviving percentage was calculated by dividing the number of CFU from plates containing TSA plus methyl viologen by the number of CFU from plates containing TSA only. Wild-type (MPAO1) and complemented (TJB10 and BH01) strains had between 10- and 14-fold increased survival on methyl viologen compared to the transposon mutant (PW4072). Values are means plus standard deviations from five independent experiments. Values that are significantly different (*P* < 0.05) from the value for the wild-type strain are indicated by an asterisk.

As low-molecular-weight thiols are important for detoxification of oxidative stressors (26), we determined whether the lack of GSH contributed to increased sensitivity to various oxidants. Indeed, no difference in sensitivity to H_2_O_2_ or to the organic peroxide cumene hydroperoxide (CHP) was observed in the absence of GSH (data not shown). However, strain PW4072 (0.003% survival) was significantly more sensitive to the superoxide generator methyl viologen than strains MPAO1 (0.032% survival; *P* = 0.04), TJB10 (0.032% survival; *P* = 0.04), and BH01 (0.041% survival; *P* = 0.01) (Fig. 9). It appears that GSH is important for detoxification of superoxides but that it plays less of a role, if any, in the detoxification of peroxides.

### Disruption of *gshA* leads to altered antibiotic sensitivity.

Since a previous study showed that a P. aeruginosa* gshB* mutant had increased sensitivity to some antibiotics (27) and other studies have found that other low-molecular-weight thiols are important for detoxification of antibiotics in Mycobacterium smegmatis (26, 28), Staphylococcus aureus (29, 30), and P. aeruginosa (31, 32), we determined the antibiotic sensitivity of the *gshA* transposon mutant. A broth dilution method was used to determine the MIC (33) of seven antibiotics using the wild-type, *gshA* mutant, and complemented strains. Strain PW4072 had increased sensitivity to kanamycin, fosfomycin, and rifampin compared to the wild-type and complemented strains. The MICs of kanamycin and rifampin for strains MPAO1 and TJB10 was 25 µg/ml, while the MIC for strain PW4072 was 12.5 µg/ml. The MIC of fosfomycin for strains MPAO1 and TJB10 was >100 µg/ml, while the MIC for PW4072 was 50 µg/ml. The MIC of rifampin for MPAO1 and TJB10 was 25 µg/ml, while the MIC for PW4072 was 12.5 µg/ml (Table 1). There was no difference with the other antibiotics.

**TABLE 1:** MICs of various antibiotics

Antibiotic	MIC (µg/ml) of antibiotic in strain:		
	MPAO1	PW4072	TJB10
Ampicillin	>200	>200	>200
Erythromycin	>200	>200	>200
Fosfomycin	>100	50	>100
Kanamycin	25	12.5	25
Ofloxacin	12.5	12.5	12.5
Penicillin G	>200	>200	>200
Rifampin	25	12.5	25

## DISCUSSION

Previously, a P. aeruginosa* gshB* mutant was found to be sensitive to antibiotics and oxidants (34). In other bacteria, such as E. coli, *gshB* mutants contain significant amounts of γ-glutamylcysteine, and this γ-glutamylcysteine has been shown to partially compensate for GSH (35). To ascertain the role of GSH without interference from γ-glutamylcysteine, transposon mutants disrupted in the *gshA* glutamate-cysteine ligase gene were characterized. Thiol analysis demonstrated that these mutants completely lacked GSH and γ-glutamylcysteine. Moreover, addition of the native *gshA* gene to one of the mutants reverted GSH levels to the level of the wild type (Fig. 1).

Like other thiol-deficient mutants, the lack of GSH in P. aeruginosa has multiple consequences. Although there is no difference in growth between the mutant and wild-type strains in enriched media (TSB), the mutant demonstrated modest growth impairment in minimal medium (M9) (Fig. 2). P. aeruginosa* gshA* mutant is also more sensitive to oxidative stress induced by the redox cycler methyl viologen (Fig. 9), but it is not sensitive to H_2_O_2_ and CHP exposure. This is in contrast to a Salmonella enterica* gshA* mutant which was not sensitive to methyl viologen (36) but was sensitive to H_2_O_2_ (12). Mutants lacking analogous low-molecular-weight thiols like mycothiol (MSH) in mycobacteria (28) and bacillithiol (BSH) in Staphylococcus aureus (37) are sensitive to H_2_O_2_, redox cycling agents like menadione and plumbagin, and the thiol oxidant diamide. Low-molecular-weight thiols are able to reduce oxidants directly or indirectly by serving as an electron donor to peroxidases (38). A GSH-dependent peroxidase (PA2826), which could potentially reduce H_2_O_2_ and/or CHP, has been identified in P. aeruginosa, although it is unclear whether the electron donor for this peroxidase is GSH (39). The lack of sensitivity to the peroxides could be due to the upregulation of proteins and enzymes involved in protection against oxidative stress similar to the upregulation of Ohr in mycobacterial thiol mutants (40–42).

Another consequence of GSH deficiency is sensitivity to the thiol-reactive antibiotics fosfomycin and rifampin. In the case of fosfomycin, a GSH transferase, FosA, which catalyzes the conjugation of fosfomycin to GSH, and in the process inactivates the antibiotic, has been reported in P. aeruginosa (31). As for rifampin, the parent compound of rifampin, rifamycin, forms *S*-conjugates with MSH in M. smegmatis (43) and BSH in S. aureus (29) and likely also forms conjugates with GSH. We did observe an increase in sensitivity to kanamycin, but not ampicillin as seen in the *gshB* mutant (34) (Table 1). Interestingly, the *gshA* mutants are also unable to form persister cells upon treatment with ofloxacin (Fig. 8). Since there is no difference in sensitivity to this antibiotic between the wild-type strain and the *gshA* mutant (Table 1), the decrease in persisters appears to be due to other factors. However, a screen for P. aeruginosa genes involved in the low-persister phenotype yielded *spoT*, *relA*, and *dksA* (44) but failed to identify genes involved in GSH metabolism.

A number of phenotypes associated with P. aeruginosa virulence are attenuated in the *gshA* mutant. Levels of the phenazine pyocyanin are lower in the *gshA* mutant, and complementation with the native *gshA* restores pyocyanin levels (Fig. 6). Pyocyanin can serve as a redox cycler similar to methyl viologen, resulting in superoxide radicals and oxidative stress. Pyocyanin can also form a GSH conjugate which is still able to redox cycle, albeit not as effectively as pyocyanin alone (45). GSH may thus serve to buffer pyocyanin within P. aeruginosa, and a decrease in GSH levels may trigger a corresponding decrease in pyocyanin.

Interestingly, Glasser et al. (46) reported that pyocyanin oxidizes excess NADPH during anaerobic metabolism in biofilms, reducing survival in hypoxic conditions at the base of the biofilm. In contrast, pyocyanin has been shown to intercalate extracellular DNA in biofilms, making the biofilm much more robust, but GSH-pyocyanin conjugates disrupt DNA intercalation, resulting in a decrease in biofilm formation (6). In MSH-containing bacteria such as Mycobacterium tuberculosis, dithiothreitol (DTT) treatment increased biofilm formation (47), and we have previously demonstrated that an M. smegmatis mutant lacking MSH (48) is impaired in biofilm formation, suggesting that redox plays a role in mycobacterial biofilm formation. In P. aeruginosa, the *gshA* mutant forms less robust biofilms, and this phenotype is reversed by the reintroduction of *gshA* (Fig. 2), indicating that GSH has some part in biofilm formation, which may be related to the decrease in pyocyanin levels in the mutant or its role as a redox modulator.

We also observed a decrease in swarming and swimming in the *gshA* mutant (Fig. 4), but not twitching. Swarming has been linked to GSH metabolism in E. coli. A mutant in E. coli CydDC, which mediates GSH transport across the cytoplasmic membrane, is impaired in swarming motility, and this motility can be restored by providing exogenous GSH (49). In addition, swarming cells have a sixfold increase in reduced GSH compared to swimming cells and a corresponding increase in resistance to oxidants (50) in *Salmonella*. Moreover, sulfur metabolism has been implicated in swarming in P. aeruginosa (7). An P. aeruginosa mutant with disrupted PA3587, which has 63% homology to *metR*, a regulator of methionine biosynthesis in E. coli, is unable to swarm. In this mutant, *metH*, encoding methionine synthase, is downregulated and the *metH* mutant is also defective in swarming.

As QS has been implicated in biofilm formation, pyocyanin levels, and swarming motility regulation, we checked to see whether AHL QS was impaired in the *gshA* mutant. Our results indicate that AHL QS appears to be qualitatively normal (Fig. 7) in the mutant. As the absolute levels of AHLs were not measured in the *gshA* mutant, it may be that these levels, like pyocyanin, are decreased but are not low enough to trigger the QS response. The alternative explanation is that *gshA* alters other quorum sensors, such as PQS, and it is these molecules that influence pyocyanin levels, swarming motility, and biofilm development.

A variety of regulatory, structural, and metabolic proteins are known to be regulated by oxidation-reduction and *S*-thiolation of cysteine residues (51, 52). In E. coli, OxyR senses oxidative stress through cysteines that are oxidized to a disulfide state, inducing the expression of antioxidant genes (53). In Bacillus subtilis, OhrR is bacillithiolated and that affects its ability to regulate antioxidant genes (54). In P. aeruginosa* ospR*, a homolog of *ohrR*, that regulates the *gpx* glutathione peroxidase gene, the oxidation of a key cysteine leads to dissociation of the protein from promoter DNA. Deletion of *ospR* leads to sensitivity to paraquat similar to the *gshA* mutant and H_2_O_2_ resistance (39). Moreover, an P. aeruginosa* oxyR* mutant (55) is unable to swarm on plates and has increased production of pyocyanin. Similar to our work, there is no difference in the AHL level in this mutant. Other regulators controlling formation of pyocyanin, swarming, swimming, and biofilm formation, could be similarly impacted by disulfide bond formation as a result of an increase in oxidative stress from lack of GSH or through the lack of *S-*glutathionylation of key cysteine residues in the *gshA* mutant. Further work is needed to identify the regulator(s) and to elucidate the mechanism of GSH control.

## MATERIALS AND METHODS

### Bacterial strains and growth conditions.

Wild-type PAO1 and all transposon strains were obtained from the University of Washington (56). The *gshA* complementation strain was generated as described below. All strains were grown in tryptic soy broth (TSB), Luria-Bertani broth (LB), or M9 minimal medium supplemented with appropriate antibiotics (60 µg/ml tetracycline and 10 µg/ml chloramphenicol for transposon mutants and 200 µg/ml spectinomycin for both TJB10 and BH01 strains). To induce expression of GshA in strain BH01, the medium was supplemented with 5 mM isopropyl-β-d-thiogalactopyranoside (IPTG).

### Complementation of strain PW4072.

*cis-*Complementation of *gshA* was accomplished by amplifying a 2-kb fragment consisting of *gshA* and the 1-kb upstream region using primers gshAcomp1 (comp stands for complementation) and gshAcomp2 (Table 2) and cloning into vector pJQ200SK using BamHI and XbaI, resulting in plasmid pTJB03. Next, the 1-kb downstream region of *gshA* was amplified using primers gshAcomp3 and gshAcomp4 and cloning it into pJQ200SK using XhoI and BamHI, resulting in plasmid pTJB06. pTJB03 and pTJB06 were digested with XbaI and BamHI, and the upstream region plus *gshA* fragment was ligated into the linearized pTJB06. The resultant plasmid (pTJB07) and pFlgB*aadA*/pCR2.1 were digested with BamHI to linearize pTJB07 and excise the spectinomycin resistance cassette. The spectinomycin resistance cassette was ligated into pTJB07 to generate pTJB08. The entire complementation region was amplified by PCR using primers gshAcomp1 and gshAcomp4. The amplicon was cloned into pCR2.1 (Invitrogen, Carlsbad, CA) according to the manufacturer’s protocol to yield plasmid pTJB10. pTJB10 was linearized using SacI and transformed into strain PW4072. Electroporation was performed as described previously (57). The *cis-*complemented strain (TJB10) was verified by PCR.

**TABLE 2:** Primers used in this study

Primer	Sequence (5′ → 3′)^a^
gshAcomp1	TCTAGAGGTCAAGGTCATGGAAGTGG
gshAcomp2	GGATCCGCCGGCTTGGCTCAGTTG
gshAcomp3	GGATCCTGATCAGCAACTGAGCCAAG
gshAcomp4	CTCGAGCACCATGCCCGACTACGTCAA
Pa5203Fptak	GCTGGGTACCTTGAGCGATCTTCTCTCC
Pa5203Rptak	GCTGGGTACCTTGAGCGATCTTCTCTCC

a Restriction enzyme sites are underlined.

*trans*-Complementation of *gshA* was accomplished using primers, Pa5203Fptak and Pa5203Rptak with KpnI and HindIII sites, respectively, to amplify the *gshA* gene from wild-type DNA. The amplified band and pJAK12 (ATCC) were restriction digested with KpnI and HindIII and ligated together followed by transformation. The transformants were screened by PCR, and plasmid DNA, pJAKgshA, from one positive transformant, pJAKgshA, was electroporated into P. aeruginosa strain PW4072 as described previously (57) to generate strain BH01.

### Thiol analysis.

Strains were grown overnight in TSB with appropriate antibiotics, and 500 µl of the overnight culture was used to inoculate 50 ml of fresh TSB. Strains were then grown to an optical density at 600 nm (OD_600_) of 1.0. Cells were pelleted and assayed for thiol production. Reduced thiols were labeled with monobromobimane (mBBr), and high-performance liquid chromatography (HPLC) analysis was performed as previously described, using glutathione (GSH) and cysteine standards to quantify thiol levels (26).

### Growth curves.

Strains were propagated in TSB or M9 medium overnight using appropriate antibiotics. The cultures were diluted 1:100 in fresh media and grown to an OD_600 _of 0.5. The cultures were then diluted to an OD_600 _of 0.05 and grown with shaking at 37°C. The OD_600_ was measured every hour, and each strain was assayed in triplicate.

### Biofilm formation.

The ability of each strain to form a biofilm was measured using a crystal violet-based assay as previously described. Briefly, strains were prepared as described above (for growth curves) and grown in a 96-well plate for 24 h at 37°C. The wells were washed three times with phosphate-buffered saline (PBS) and incubated with 0.4% crystal violet for 15 min. The wells were then washed three times with PBS, and biofilms were solubilized using 33% (vol/vol) acetic acid. Biomass was measured by reading the OD_570_. Each strain was assayed in triplicate.

### Motility.

Swarming (0.8% nutrient broth, 0.4% agar, 0.5% glucose), swimming (0.8% nutrient broth, 0.3% agar, 0.5% glucose), and twitching (0.8% nutrient broth, 1.0% agar, 0.5% glucose) plates were used to assess motility. For swarming and swimming, 3 µl of overnight cultures grown in TSB plus antibiotics were spotted onto plates that had been air dried for 30 min. For twitching, the overnight culture was stabbed through the agar. The plates were incubated for 24 to 48 h at 30°C. Motility was assessed by measuring the diameter of the widest point of spread on each plate. Each strain was assayed four times.

### Pyocyanin production.

Overnight cultures were diluted to an OD_600_ of 1.0, and 250 µl was used to inoculate 25 ml of TSB. Pyocyanin production was assayed as described previously (58). Briefly, cultures were incubated at 37°C with shaking for 18 h and centrifuged, and the resultant supernatant was then used for pyocyanin quantification. In brief, 600 µl of chloroform was added to 1 ml of supernatant, and the tube was vortexed twice for 10 s. The tubes were centrifuged at 10,000 rpm for 10 min, and the bottom phase (600 µl) was transferred to a new tube containing 300 µl of 0.2 N HCl. The tubes were vortexed twice for 10 s each time and centrifuged at 10,000 rpm for 2 min. The OD_520_ of the top phase was measured and multiplied by 17.072 to calculate the micrograms of pyocyanin per milliliter. All strains were tested four times.

### Acyl homoserine lactone-dependent quorum sensing.

Quorum sensing (QS) was testing using a cross-feeding assay with the E. coli MG4 reporter strain containing pKDT17 and the P. aeruginosa strain being tested (59). Briefly, overnight cultures of the test P. aeruginosa strains were streaked 0.75 cm from *E*. *coli* MG4 on MacConkey agar plates. The plates were incubated overnight at 37°C. Strains were classified as positive for QS when the reporter E. coli strain turned a purple/pink color.

### Determination of MICs for antibiotics.

Antibiotic sensitivity of the strains was determined as previously described (60, 61) with slight modifications. Briefly, the individual antibiotics were serially diluted in M9 medium in a 96-well plate. Overnight cultures of the individual strains were diluted 1:100 in fresh M9 medium and grown to an OD_600_ of 0.5. The culture was then diluted to an OD_600_ of 0.05 in fresh M9 medium and added to each well of the 96-well plate containing antibiotics, giving a final OD_600_ of 0.025. The plate was incubated with shaking at 37°C for 18 h. The MIC was determined as the lowest concentration of antibiotics with no visible cell growth following incubation. Each antibiotic was tested against each strain four times.

### Persister cell formation.

Persister cells were formed as described previously (62) with slight modifications. Briefly, P. aeruginosa was grown for 18 h in TSB. Cells were pelleted by centrifugation and washed three times with 0.85% NaCl. The cells were resuspended in 0.85% NaCl and split into two cultures. One culture was treated with 50 µg/ml of ofloxacin, and the other culture was left untreated. The cultures were incubated at 37°C for 3.5 h before being washed three times with 0.85% NaCl. The cells were resuspended in 0.85% NaCl, and serial dilutions were plated on tryptic soy agar (TSA) for overnight incubation. Persister cell formation was determined by comparing the CFU of the ofloxacin-treated cells and untreated cells and calculating the percentage.

### Determination of oxidant resistance.

Oxidant resistance was determined as previously described (63) with a few modifications. Briefly, overnight cultures were grown in TSB and diluted the next morning using fresh medium. Cells were grown to exponential phase and serially diluted in PBS. Each dilution was spotted (10 µl) on TSA plates alone or with 1 mM methyl viologen, 0.5 mM H_2_O_2_, or 1.5 mM cumene hydroperoxide (CHP). The plates were incubated overnight at 37°C, and ensuing colonies were counted. The surviving fraction was calculated by dividing the number of cells grown on the plate containing oxidant by the number of cells grown on the plate without an oxidant. Each strain was tested in triplicate.

### Statistical analyses.

All data were analyzed using a one-way analysis of variance (ANOVA) followed by *posthoc* comparisons using the Tukey test in GraphPad Prism 6.
